# NEIBank: Genomics and bioinformatics resources for vision research

**Published:** 2008-07-18

**Authors:** Graeme Wistow, Katherine Peterson, James Gao, Patee Buchoff, Cynthia Jaworski, Catherine Bowes-Rickman, Jessica N. Ebright, Michael A. Hauser, David Hoover

**Affiliations:** 1 Section on Molecular Structure and Functional Genomics, National Eye Institute, National Institutes of Health, Bethesda, MD;; 2 Department of Ophthalmology, Duke University Medical Center, Durham, NC;; 3 Department of Medicine, Duke University Medical Center, Durham, NC;; 4 Helix Group, Center for Information Technology, National Institutes of Health, Bethesda, MD

## Abstract

NEIBank is an integrated resource for genomics and bioinformatics in vision research. It includes expressed sequence tag (EST) data and sequence-verified cDNA clones for multiple eye tissues of several species, web-based access to human eye-specific SAGE data through EyeSAGE, and comprehensive, annotated databases of known human eye disease genes and candidate disease gene loci. All expression- and disease-related data are integrated in EyeBrowse, an eye-centric genome browser. NEIBank provides a comprehensive overview of current knowledge of the transcriptional repertoires of eye tissues and their relation to pathology.

## Introduction

The successes of large scale genome projects provide tremendous opportunities for understanding biology and pathology at the molecular level. Yet the mass of genomic data also presents huge challenges in extracting and organizing data relevant to specific research objectives. NEIBank [1,2] is a project for ocular genomics that provides data resources and analytic tools for gene discovery and investigation of gene expression in the eye. It began with the construction and expressed sequence tag (EST) analysis of a series of cDNA libraries made from dissected human eye tissues [1,3-6] and has expanded to cover eye tissue cDNA libraries for many model species ranging from mouse and dog to chicken and zebrafish ( [7-16] and unpublished data), in addition to other sensory tissue libraries from ear and taste cells. The sequence verified cDNA clones picked in the EST analysis are available to investigators for expression and structure/function studies.

EST analysis determines the partial sequence of cDNA clones. The next step in the analysis is to determine the gene from which a particular sequence is derived. This process relies on a combination of identification by sequence identity, overlap with known genes, genome position of the sequence, and quality control measures to identify and filter out erroneous or artifactual data. The catalog of genes identified by EST sequencing of an unnormalized, unsubtracted cDNA library reflects a random sample of the mRNA present in the cell. At least for abundantly expressed genes, the frequency of cDNAs from a specific gene provides an indication of the relative level of transcription of that gene. The NEIBank project has developed bioinformatics tools to identify, organize, and annotate these EST data and those from other eye- and ear-derived sequence data sets deposited into the dbEST/Unigene [17] sections of GenBank. NEIBank provides a comprehensive database of the EST-based expression data for the tissues of interest.

The NEIBank database has expanded beyond cDNA collections to include data for serial analysis of gene expression (SAGE) studies of expression patterns in human retina and retinal pigment epithelium, assembled in EyeSAGE [18]. In addition, comprehensive, annotated, and updated databases of known and candidate eye disease genes and chromosomal regions have been assembled. All these data are integrated through Web-based tools—particularly through a powerful eye-centric genome browser, EyeBrowse, that displays eye-related expression data for human and model species in the context of the latest genome builds.

NEIBank is a unified resource for understanding what is known about gene expression in the eye, providing tools for identifying cDNA clones for functional studies, and examining the genomics of human eye disease. Here we describe major additions and enhancements to NEIBank implemented since it was first described in Mol Vis in 2002 [1,2].

## Methods

### Analysis of EST data

**Figure 1:**
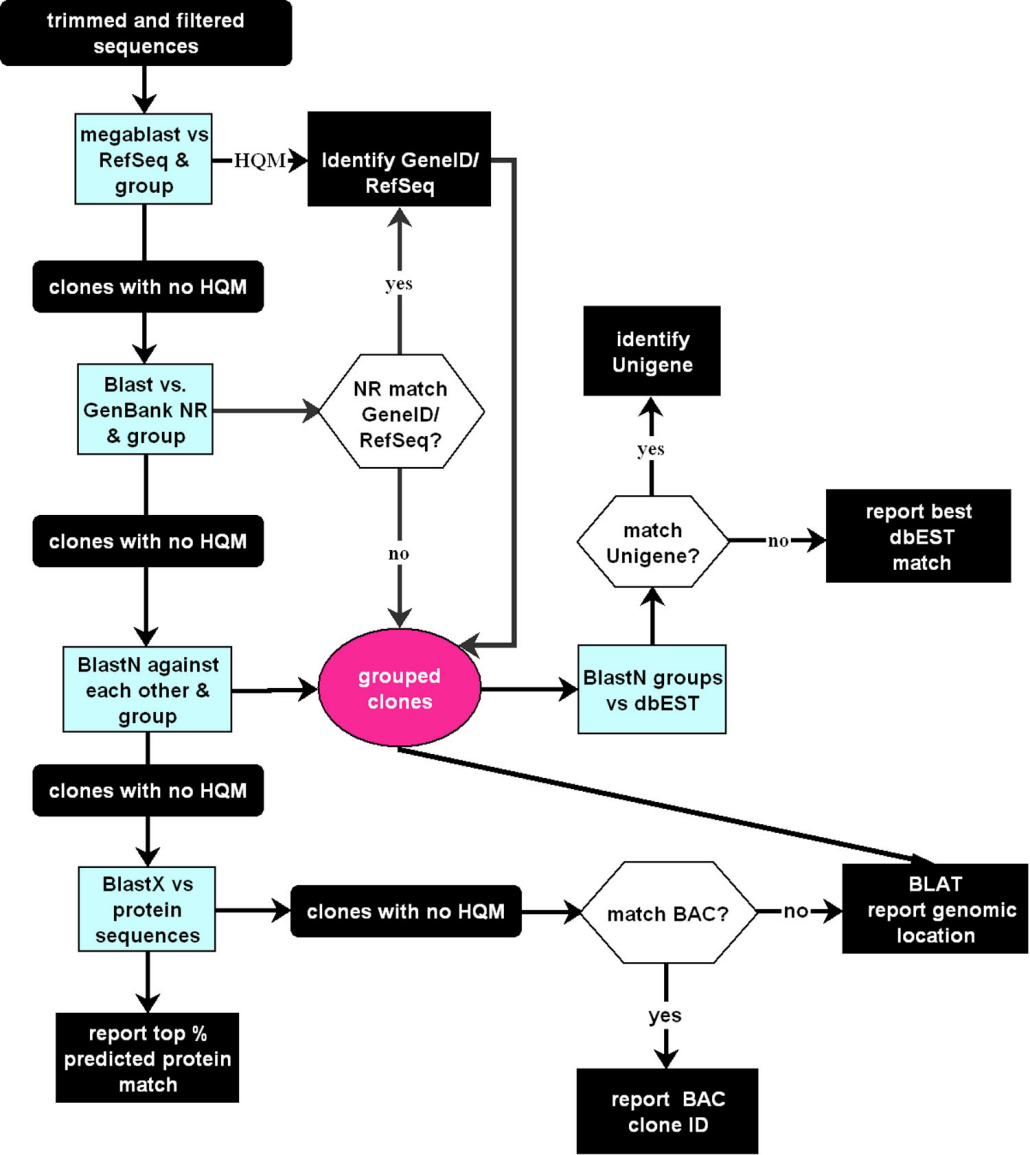
Flowchart for GRouping and Identification of Sequence Tags (GRIST). High quality matches (HQM) under our default conditions are at least a 97% identity over a minimum length of 50 bp for NCBI RefSeq/NR (non-redundant) database matches and 96% identity over a minimum 100 bp length for NCBI dbEST database matches. Blast matches against NR are filtered to ignore multigene clones (such as bacterial artifical chromosomes [BACs]) and known artifacts. NR matches are checked for GeneID and are grouped with RefSeq matches for the same GeneID. This takes account of short or incomplete RefSeqs. Unigenes are assigned independently by BLAST against dbEST. UniGene assignments for the top eight HQM dbEST matches for each clone are identified, and those that occur at frequencies of at least 15% for the whole group are reported. This can help identify Unigene problems, overlapping genes, and variant transcripts.

EST data for NEIBank are analyzed and annotated using a procedure called GRIST: **GR**ouping and **I**dentification of **S**equence **T**ags. GRIST uses a rule-based procedure mainly reliant on basic local alignment search tool (BLAST) [19,20] searches of National Center for Biotechnology Information (NCBI) DNA and protein databases [21] together with self-match searches of the clones in each library to assemble clusters or groups of ESTs corresponding to specific genes and to identify them independently through matches to GenBank and UniGene. GRIST also annotates clusters of cDNAs with Gene Ontology (GO) [22] terms and chromosomal and genomic location. Since its original description, GRIST has been implemented in Oracle 9i (Oracle Corporation, Redwood Shores, CA: a commercial structured query database implemented in Unix at NIH) and has been updated and modified to improve functionality and speed of processing. In particular, links to LocusLink (which has been discontinued) have been replaced with links to Entrez Gene; primary identification with full-length cDNA sequences now uses the NCBI human, mouse, and “other” reference sequence (RefSeq) databases (which speeds processing; both high level and detailed GO terms extracted from Entrez are included for functional annotation. The current GRIST pipeline is shown in Figure 1.

### Chimera search

The sequences of individual clones are aligned to genome builds using BLAST-like alignment tool (BLAT) a tool for rapid alignment to genome sequences [23]. Clones with high quality matches (98% identity over at least 50 bp) for different regions of their sequence on different chromosomes or on the same chromosome with spacing over 100 kb are flagged as potential chimeras (artifact cDNAs that result from the fusion during cloning). These are verified manually. This process screens most chimeric cDNA clones out of the EST analysis. Since GenBank itself also contains chimeric sequences derived from one or more genes, the NEIBank database maintains a list of known problem-sequences. BLAST matches to these are ignored by GRIST.

### Library comparison tool

Libraries for comparison are selected from a menu page. For each cluster of clones, the equivalent cluster (if any) in the other library is located and abundance (clone count and frequency in percent) data for both libraries are displayed. Libraries from the same species are compared using Entrez Gene ID. Libraries from different species can be compared if there is an available mapping of the orthologous UniGenes through Homologene. Tab delimited text output is available.

**Table 1:** NEIBank Custom cDNA libraries used for EST analysis.

**Human**	**Rabbit**
iris (bx) NbLib0017	cornea (naf) NbLib0086
iris normalized (fg) NbLib0016	eye minus lens and cornea (nag) NbLib0087
keratoconus cornea (od,oe) NbLib0073	lens (nbd) NbLib0112
lacrimal gland (oj) NbLib0076	trigeminal nerve (nbc) NbLib0114
lens (by) NbLib0019	
lens normalized (fs) NbLib0020	**Guinea pig**
optic nerve (nbj) NbLib0119	eye minus lens and retina (nba) NbLib0109
pterygium (nav) NbLib0106	lens (nbb) NbLib0111
retina (hd,he) NbLib0042	retina (naz) NbLib0110
retinal pericyte (hw,hx) NbLib0038	
RPE/choroid (cs) NbLib0047	**Chicken**
Retina Y2H* (nbp) NbLib0129	embryo eye (nax) NbLib0105
	hatched eye (naw) NbLib0104
**Mouse**	
lacrimal gland (ou) NbLib0081	**Zebrafish**
lens Y2H* (nbf) NbLib0113	anterior segment (nap) NbLib0092
retina (mk,ml) NbLib0058	lens (nab) NbLib0085
retina Y2H* (nbk) NbLib0120	posterior segment (nao) NbLib0095
RPE/choroid (mi,mj) NbLib0059	retina (naq) NbLib0093
whole eye (io,ip) NbLib0032	whole eye (naa) NbLib0084
No3 whole eye (ob,oc) NbLib0074	
	**Others**
**Rat**	iguana lens (hm) NbLib0005
eye angle (gw,gx) NbLib0041	kangaroo lens (mw) NbLib0056
lens (nar) NbLib0094	echidna whole eye (ot) NbLib0082
retina (hf,hg) NbLib0063	cow lens (nbm) NbLib0125
whole eye (jd,je,kr,ks) NbLib0061	rhesus monkey lens (nbl) NbLib0126
	mouse organ of corti (gi) NbLib0053
**Dog**	human fetal cochlea (n) NbLib0010
cornea (nad) NbLib0089	mouse taste cell (ia) NbLib0123
eye minus lens (or) NbLib0080	lens (nac) NbLib0088
eye minus lens and cornea (nae) NbLib0090	

This table lists the custom cDNA libraries used for EST analysis for NEIBank. Each library has a database number (e.g. NbLib0017) and code letters (e.g. od,oe) that indicate clone ID prefixes derived from the high throughput sequencing pipeline. Asterisks indicate that the library was constructed for yeast two hybrid (Y2H) screening. An additional human retinal pigment epithelium and choroid Y2H library has been constructed but has not yet been used in expressed sequence tag (EST) analysis.

### EyeSAGE

The EyeSAGE [18] database contains SAGE expression data for human retina and retinal pigment epithelium (RPE), derived from new SAGE libraries and from previously published human retina and RPE data extracted from SAGE Genie [24], and combined data for neural and other tissues for comparative purposes. The original EyeSAGE database, in Excel format [18], is available at EyeSAGE. These data were imported into NEIBank, and tag assignments are updated from the latest release of SAGE Genie. Unigene, Entrez*,* and other annotations are also added and updated.

### Eye disease databases

The NEIBank database was assembled by mining Online Mendelian Inheritance in Man (OMIM) under several categories of genes that included a “clinical synopsis” with terms under “Eye.” Other disease genes and mapped disease gene regions were extracted from OMIM by manual searches for specific eye disease terms (such as cataract or glaucoma). Completeness for retina diseases was checked by comparison with RetNet. Other genes and disease regions were identified using PubMed literature searches.

Genomic regions linked to particular eye diseases were entered into the database with their published sequence tagged sites (STS) markers. These were mapped onto the current human genome build using the UCSC Genome Browser to define the disease region in nucleotide positions. Many published STS markers could not be located in the genome build, and alternatives had to be manually substituted using UniSTS or by locating other markers based on cytological bands or mapped genes.

The disease gene database is organized by chromosomal location and contains either OMIM or PubMed links for both the gene and the corresponding disease as well as links to NEIBank clone information, GenBank, Entrez Gene, UniGene, and Entrez single nucleotide polymorphisms (SNPs). Each entry has been manually annotated for broad categories of eye tissue affected and disease class, which can be used to search the database.

### EyeBrowse

The EyeBrowse is a customized eye-centric version of the UCSC Genome Browser browser. Custom tracks for eye-derived ESTs from all species with available genome builds are included. Additional tracks show eye disease genes, candidate eye disease regions, and EyeSAGE data. SAGE data are displayed as histograms scaled to the represent the total normalized tag count from eye tissues [18]. The bars are hyperlinked to complete EyeSAGE data for each gene, including comparison data for tissues from sources other than eye.

### Updates

Genome builds are updated in step with the UCSC Genome Browser site, and current versions are indicated on EyeBrowse and disease gene pages. GRIST updates are performed periodically in response to significant changes in NCBI Unigene and RefSeq databases. This varies by species but typically each library data set is completely updated twice a year. SAGE tags are similarly updated from SAGE Genie. A Summary page lists all data sets, their update dates and statistics.

### Array data

At present NEIBank does not include data from microarray experiments. We have not yet identified any rigorous way to display such data that doesn’t simply duplicate Gene Expression Omnibus (GEO).

## Results and Discussion

### NEIBank EST data

High throughput sequencing of cDNA libraries by EST analysis has been a powerful technology to identify the transcriptional repertoire of tissues, to identify alternative transcripts and to provide resources of clones for other experiments. So far, about 50 custom cDNA libraries covering 13 species have been created and analyzed for NEIBank [2-10,12-14,16] (Table 1). Many previously uncharacterized genes and variants have been discovered and reported. Recently an NEIBank EST from human lens helped identify a novel lens-preferred gene as the locus for an inherited human cataract [25], while data for rabbit eye tissues have been used to construct a novel rabbit DNA microarray for expression studies [14].

**Figure 2:**
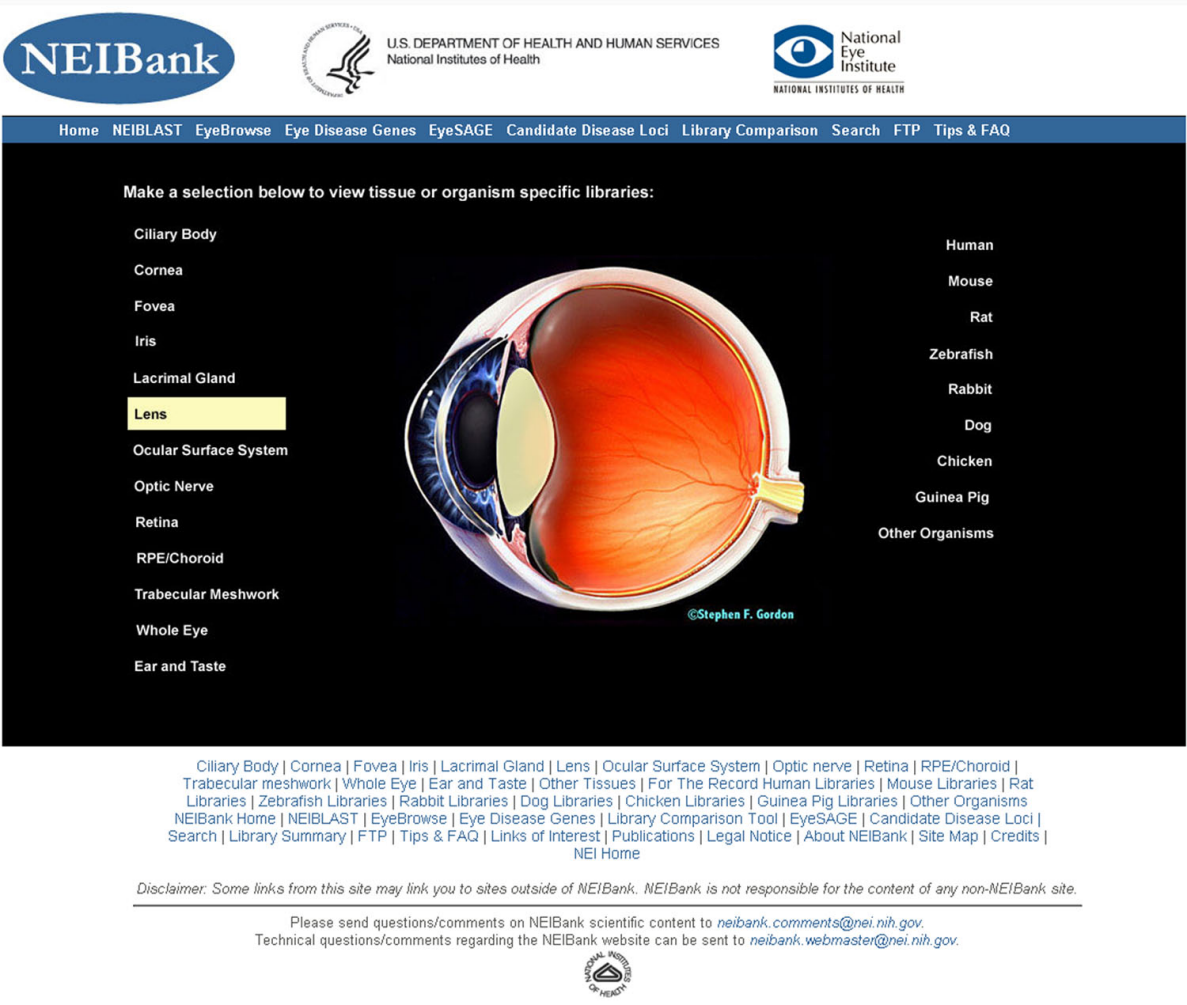
NEIBank home page. Data on specific tissues can be selected by text or image links (here lens is selected). The page also contains text links to data organized by species and to other databases and informatics tools.

**Figure 3:**
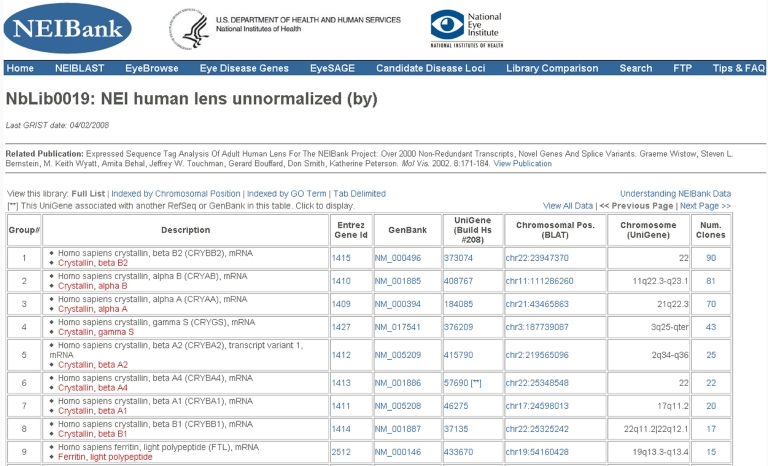
Typical library summary page. This Web page displays the analyzed EST data from a lens-derived cDNA library. Clones are ranked in order of abundance but can also be displayed and sorted by chromosomal location or by functional keyword (GO terms).

In addition to the organized and curated display of the EST data available at the NEIBank website, sequence data from all NEIBank libraries are deposited in GenBank. At the same time, the NEIBank database extracts data from other eye-related cDNA libraries in GenBank, specifically through the NCBI Unigene database.

Since eye tissues share functional and pathological connections with other sensory tissues, NEIBank also hosts EST data for human and mouse ear cDNA libraries (see for example [26]) in collaboration with the National Institute of Deafness and Other Communications Disorders (NIDCD). In particular, this allows comparison of expression data in eye and ear that may be relevant to conditions such as Usher’s syndrome [27,28]. Among recent additions to NEIBank, also in collaboration with NIDCD, are data from another sensory cell type: mouse taste receptors.

NEIBank uses a procedure called GRIST (Figure 1) to identify and group or cluster EST sequences. This provides reliable clustering and identification of clones. Some clones from introns or flanking regions of a particular gene may match the Unigene for that gene but do not group in GRIST. Rather than force these ambiguous clones into groups, they are listed separately. Groups that share Unigene identifiers are indicated by [**]. Clicking on this symbol runs a search to show them all. The possible identity of other clones with poor sequence is indicated by predicted protein match.

Artifacts can commonly arise through the process of cDNA cloning, often through ligation of different cDNAs or through mutual priming of different first strand cDNAs during second strand synthesis. This produces chimeric or fused cDNA clones that cause problems for clustering methods and are unlikely to be useful as resources for expression studies. For NEIBank, chimeric ESTs are identified by ChimeraSearch and by manual curation and are then removed.

Data from libraries made especially for NEIBank (designated as “NEI” libraries on the website) have the highest level of curation. The data from other EST analyses retrieved from UniGene are designated as “dbEST” libraries. In some cases data from several related libraries have been pooled for convenience. Each web page for combined dbEST data indicates the UniGene Library Identifiers for of each constituent library. Dates and statistics (numbers of ESTs and number of identified clones) for all library data sets are listed on the Library Summary page.

### Browsing EST data at the NEIBank website

NEIBank generates web pages for each of the GRIST-analyzed cDNA libraries. These can be accessed from the home page (Figure 2), which has an image of the human eye and hyperlinks and text links to data for different eye tissues and species. Each library page has a common format (Figure 3). The first line provides the library name and description including the source of the cDNA, the species, the tissue, and, for NEIBank-generated cDNA libraries, a letter code (such as *by* or *he*, derived from the high throughput sequencing pipeline) that catalogs the libraries or sublibraries included. The next row provides references and links to publications describing library construction and analysis. The table header also provides user-selectable options for sorting, viewing, and formatting the data table. The default web page view of each EST collection is organized according to the abundance of clones in each group in descending order. For each cluster of clones, there is a description field that contains two identifiers: one derived from RefSeq (or GenBank NR) matches and the other from UniGene matches. While RefSeq/NR and UniGene identifications usually agree, this is not always the case. Including independent assignments of identity increases the chances of finding more informative names and also reveals potential issues arising from undetected chimeras or from overlapping genes.

**Figure 4:**
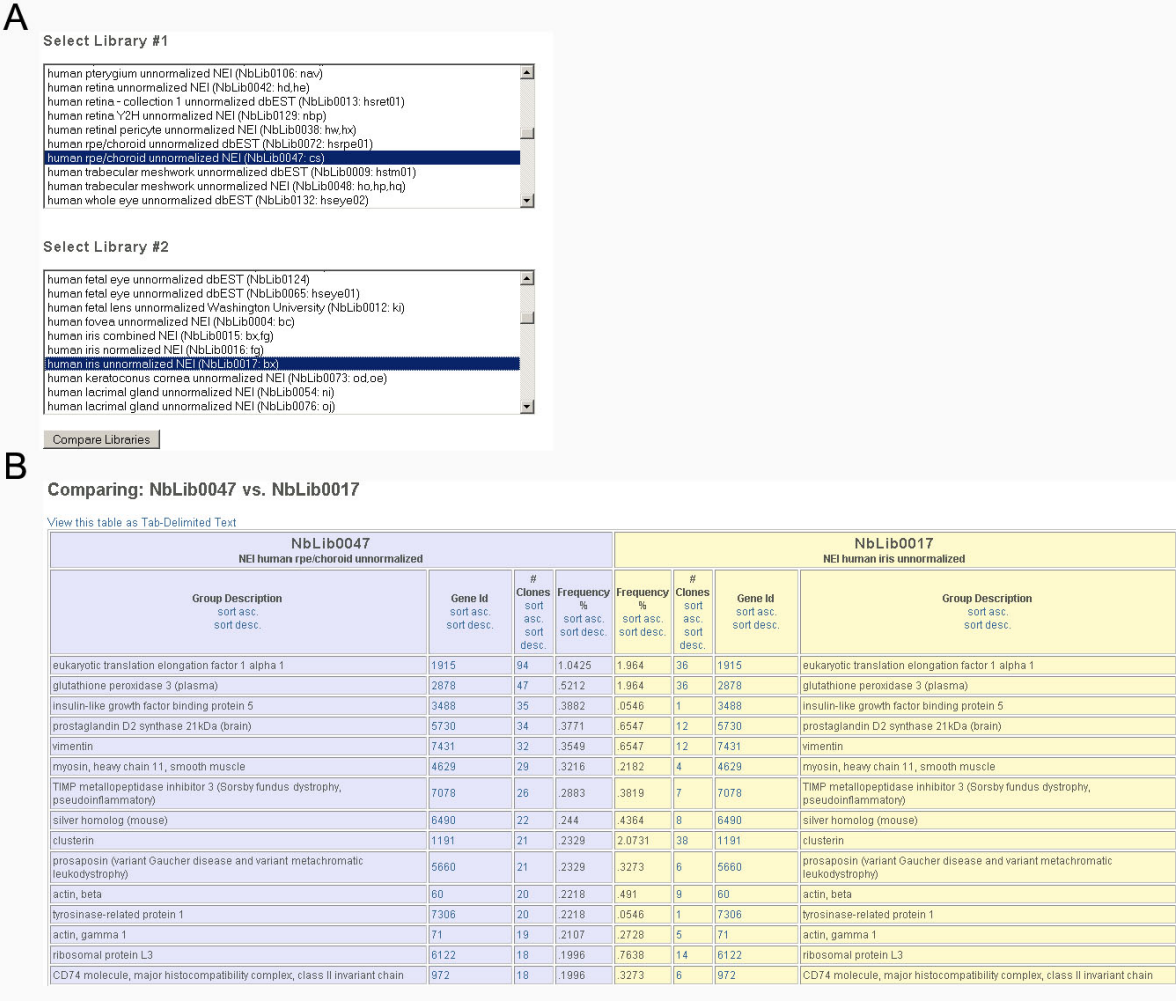
Library comparison. **A:** EST data for pairs of cDNA libraries can be selected for comparison. **B:** The output lists the genes identified in the first library, ranked by number and frequency of ESTs, and shows the number and frequency of ESTs for the same gene in the second library. This provides useful comparisons for relatively abundant transcripts in unnormalized (native abundance) cDNA libraries. For the same species (e.g., human retina versus human retinal pigment epithelium), libraries are compared based on common Entrez Gene IDs. Different species for which orthologous GeneIDs are linked through Homologene, can be compared (e.g., human retina versus mouse retina).

Linked Entrez Gene ID, RefSeq or NR (from sequence match) and UniGene identifiers are also shown for each cluster, providing direct access to information about each of the genes. The position in the genome of a representative clone in each cluster (from BLAT alignment) is also shown and links to the EyeBrowse genome browser. For human and some other species the cytological band nomenclature (extracted from Entrez Gene) is included. Occasionally there are multiple UniGenes listed for a single cluster of clones. This arises from undetected chimeras, duplicated genes, highly conserved pseudogenes, or from overlapping genes (for example, protocadherin genes have multiple Gene ID/UniGene designations for transcripts with common 3′ ends). Some entries show more than one chromosomal position. Often this arises from the presence of highly conserved pseudogenes in the genome, some of which may be functional.

The last column of the library summary table shows the total number of clones in the cluster, hyperlinked to a page that lists the clone IDs (in 96-well plate format) with links for ordering specific clones. In turn, this page links to a FASTA format display of the EST sequences with further links for BLAT alignment of those sequences on selected genome builds. For all cDNA libraries, tab delimited text output is available and complete FASTA formatted sequence collections can also be downloaded from the FTP site.

From the main library pages EST data can also be listed by chromosomal location or by GO terms. The GO annotations are extracted from Entrez Gene and can be viewed as high level or detailed terms. For mouse data only, an additional link is included to the GeneNetwork/WebQTL database. This displays a tabulation of the relative expression levels for each gene in multiple strains of laboratory mice obtained from large scale microarray analyses [29].

### Searching NEIBank

Specific clones or annotated genes can be located using the Search page. Individual NEIBank clones can be located either by their GenBank accession number or by their NEIBank clone ID. Clones for particular genes can be selected by the accession number of RefSeq or NR match or by text description (such as “crystallin” or “opsin”). A homology search identifies orthologs in other species (as defined by Homologene) of a human gene of interest. GO terms can be searched using wild cards (such as *synap*) to retrieve multiple, nonoverlapping relevant GO terms (this is often much more useful than searching by individual specific terms). Searches may be made on the complete database or limited by species, tissue, or other terms. The EST databases of NEIBank can also be searched by BLAST through the NEIBLAST page using query DNA or protein sequences. Each matching sequence is linked to its cluster in NEIBank through an automatic search.

### Comparing EST data

Pairs of libraries can be compared using the Library Comparison Tool, which is linked from the NEIBank home page. When two data sets are selected (Figure 4A), the output (Figure 4B) displays a listing in the order of abundance within the first library with the corresponding data (based on identical Entrez Gene ID or equivalent from Homologene) for the second library. The number of clones for each library is shown along with the “frequency %” of the clones in the library. This can give useful comparative information for the most abundant clones. For example, striking differences in the transcriptome can be seen in comparisons of human and mouse libraries for retina, RPE/choroid, lacrimal gland, among others [2]. This may be an important consideration in choosing model species for studies of human eye diseases. The presence or absence of low abundance transcripts in EST data sets is essentially stochastic, so it is important to remember that absence of a gene among the sequenced clones does not mean it has no expression in the tissue of interest. Furthermore, comparisons are also only meaningful for pairs of unnormalized, unsubtracted libraries for which cDNA abundance can reflect mRNA abundance (most NEI libraries were deliberately constructed in this way and so fit this definition). Note also that useful comparisons can only be made for species with Entrez Gene ID as well as Unigene data, and that cross-species comparisons can only be made for pairs of species for which orthologous genes have been identified.

### EyeSAGE

Eye expression profiles in NEIBank have been expanded by incorporation of EyeSAGE data [18]. SAGE data are based on short tags derived from the 3′ end of cDNA clones that are concatenated and sequenced. Tags can be identified with particular genes and counted to give a measure of the relative expression levels of different genes. Combining EST and SAGE data gives a broader view of the transcriptome in a tissue.

The NEIBank update of EyeSAGE can be queried directly. Specific genes can be looked up by gene name, Entrez Gene ID, accession numbers, text, or GO terms. The returned information includes the gene name (linked to the eye-centric EyeBrowse genome browser); a link to details of the SAGE tags assigned to that gene; an automatic search of Entrez gene ID; UniGene ID; and a link to search the SNP database for that gene. The linked listing of tags for each gene shows the individual normalized tag counts for each of the human retina and RPE SAGE libraries, along with normalized counts for Eye Sum (sum of all eye SAGE libraries), Neural Sum (sum of all libraries from neural tissues), and Body Sum (sum for all other body tissues) to give an indication of tissue preference. Most of the SAGE data currently available are based on short tags. In the NEIBank database, tags from the EyeSAGE long tag library are always displayed in association with the corresponding short tag. In some cases the SAGE Genie assignment of the long and short tags designates different genes, since assignment of some SAGE tags to specific genes is still evolving. If so, both tags are then listed under both genes by NEIBank until the issue is resolved in SAGE Genie.

**Figure 5:**
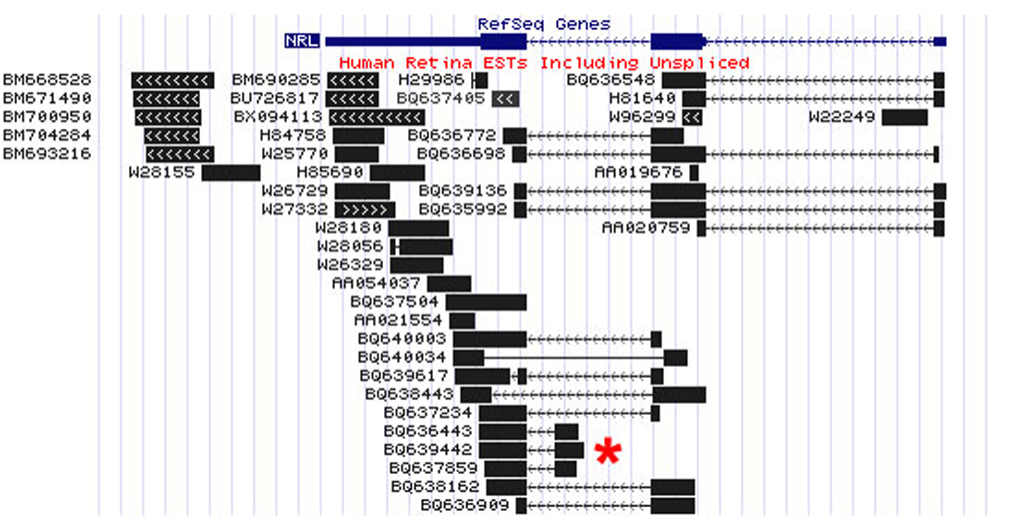
Detail of EyeBrowse view of ESTs for the human NRL gene. ESTs for NRL from human retina are shown aligned with the RefSeq gene. The red asterisk highlights a group of three cDNAs, all of which contain an alternative exon with a potential protein coding open reading frame. Note, also, there are several ESTs from a longer 3′ UTR of the gene that do not overlap with the RefSeq sequence.

### Eye disease gene databases

Many genetic mutations and sequence variants acting in both Mendelian and quantitative trait (QTL) fashion can affect vision. New alleles and candidate disease regions are being identified by techniques such as haplotype SNP identification and genome-wide association studies. Recent examples of this include the association of a common complement factor H variant with risk of age-related macular degeneration [30-34] and the use of population studies, such as the Beaver Dam Study, to look for genetic susceptibilities to common conditions such as age-related cataract [35]. Identification of disease genes opens up possibilities both for diagnostics and therapeutics.

**Figure 6:**
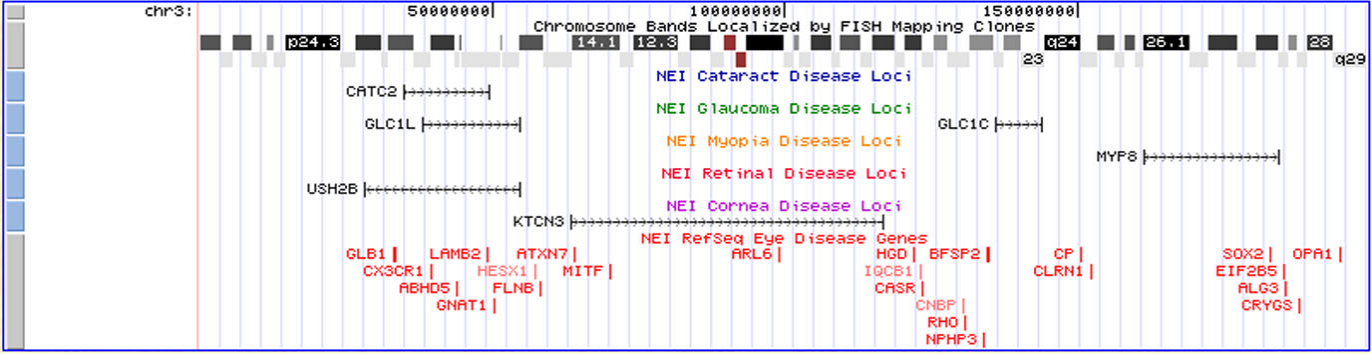
Eye disease genes and candidate disease tracks. An overview of human chromosome 3 shows the positions of known eye disease genes and candidate eye disease region tracks (with gene and expression tracks toggled off). Approximately 200 Mb of sequence is displayed. The chromosome banding pattern is illustrated followed by the regions that display positive linkage for various eye diseases. Displayed below the linkage regions are the genes annotated by the NEIBank Disease Gene database.

RetNet provides an excellent online resource for genetically based retinal diseases, but at this time, no single, comprehensive listing of eye disease genes is widely available on the Internet. Although OMIM is an important resource for disease gene information, it is not easily queried to produce a defined and complete list of eye diseases. OMIM, RetNet, and PubMed were used to assemble a database for NEIBank of genes (438 as of this writing) that affect vision. A subsidiary database lists other genes (currently 165) with more general cranial facial or neuromuscular associations with the eyes (such as blepharospasm or hyperteleorism) or genes with broad systemic effects that may not affect vision directly. Another table lists eye diseases related to chromosomal aberrations. The genes in the NEIBank Eye Disease database are also displayed on EyeBrowse as the “NEI RefSeq Eye Disease Genes” track.

Each “disease gene” has also been manually annotated for broad categories of affected tissue and disease type (e.g., retina, cornea, macular degeneration, or cataract). The database can be queried by these terms—for example, producing a list of cataract-associated genes.

**Figure 7:**
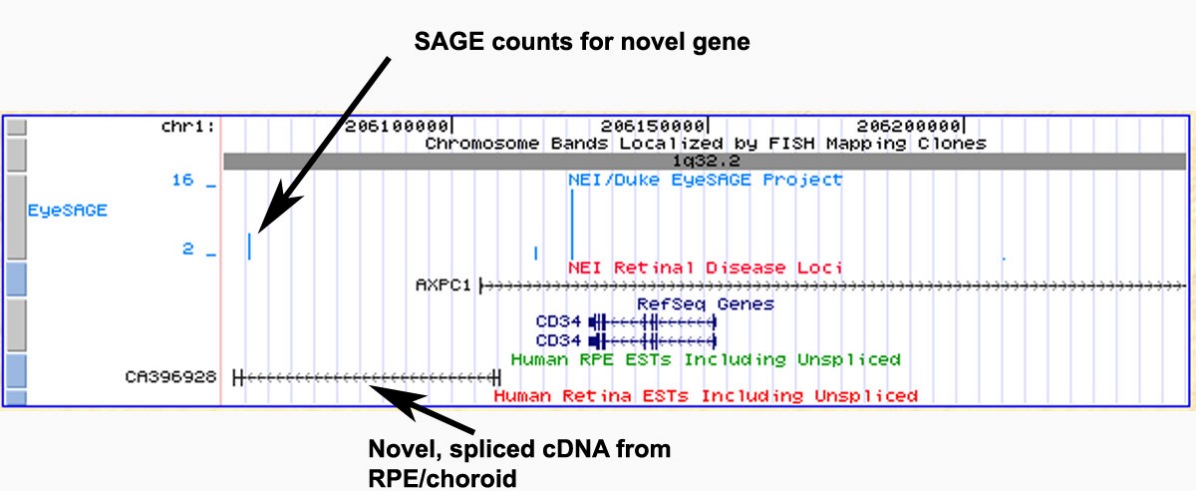
Part of a candidate disease region (AXPC1) showing a novel spliced gene transcript. This view shows a detail of one end of the AXPC1 region. An expressed sequence tag (EST) from the retinal pigment epithelium (RPE) shows the structure (vertical bars are exons; arrowed lines show introns) of a novel gene. EyeSAGE has tag counts for some Unigenes in this region, including the novel gene (designated as C1orf132 in Unigene). Note that the EyeSAGE tag bars are positioned according to the corresponding Unigene. Their positions may change slightly with different releases of Unigene.

Mapped candidate chromosomal regions for several categories of eye disease (retinal disease, cataract, myopia, glaucoma, and corneal disease) have been collected from OMIM and PubMed and incorporated into the NEIBank database. These regions can be displayed in table form with genome positions and links to other databases. The name of each disease is linked to OMIM, where possible, or to PubMed if there is no current OMIM entry. For retinal and RPE diseases, the EyeSAGE [18] data for expressed genes in the interval are also listed. Alternatively, the candidate disease regions can be displayed graphically as tracks in EyeBrowse, as described in the following section.

So far, no automated method for updating the databases of disease genes and regions exists, so input of additions and corrections from the eye research community is encouraged. A convenient contact method is email to NEIBank comments.

### EyeBrowse

EST, SAGE, and disease databases are integrated in EyeBrowse, a customized eye-centric version of the UCSC Genome Browser. Several standard UCSC tracks are included in EyeBrowse—for example, those for chromosome bands, RefSeq, and small RNAs. The AceView [36] track is also included and is useful for searching candidate disease regions. AceView is essentially a compilation of all known splice variants of expressed genes and represents rare transcripts (some of which are noncoding) that are not included in RefSeq. Like the UCSC Genome Browser, EyeBrowse can be queried in various ways with gene names, accession numbers, sequence ranges, or STS markers. Different tracks can be toggled on or off and their data shown at different densities (in most cases the best option for viewing EST and RefSeq tracks is “pack”).

EyeBrowse displays EST data tracks specifically for eye-derived cDNA libraries, organized by tissue (for example, human lens or retina). In addition to human, EyeBrowse hosts data for all species for which both eye EST and genome sequence are available through the UCSC genome builds; currently this includes mouse, rat, dog, cow, rhesus monkey, chicken, and zebrafish. EyeBrowse thus provides a convenient way to display all the known eye-expressed ESTs in and around a particular gene. This provides a rapid graphical overview of EST-based expression patterns and alternative transcripts for particular genes and allows easy identification of potential full-length clones for expression studies. For example, Figure 5 shows human retina EST data for the important retinal transcription factor NRL [37]. The asterisk indicates three aligned clones for an alternative transcript of this gene using an additional exon. The alternative exon contains an open reading frame and is also observed in the orthologous mouse gene (not shown), suggesting that it may have functional significance [2,4].

EyeBrowse also displays EyeSAGE data [18] as a custom track. Each SAGE tag is indicated by a bar centered on the midpoint of the alignment of each Unigene on the current version of the human genome. (Note that for some genes, the existence of unusual, long transcripts in Unigene may move the defined midpoint away from the mid point of the RefSeq gene position.) The height of the bar corresponds to the total count of tags observed in all the eye-derived SAGE data sets. Clicking on the EyeSAGE bar brings up a full listing of gene and SAGE tag details from the NEIBank EyeSAGE database.

Another custom track displays the RefSeq positions for the genes that affect vision, taken from the NEIBank human eye disease gene database. Other custom tracks display the extent of the candidate disease loci arranged by broad disease categories, such as “retina disease,” “cataract,” or “myopia.” Figure 6 presents an overview of chromosome 3 showing the cataract, glaucoma, myopia, and retina disease tracks with several overlapping disease regions. For this overview, other tracks are toggled off. For detailed views, the image can be zoomed in and other tracks added to display gene expression data.

The overview of eye expression data in disease loci provided by EyeBrowse can help in prioritizing candidate genes and in identifying potentially novel genes in those regions. For example, Figure 7 shows part of the region mapped for Posterior Column Ataxia with Retinitis Pigmentosa (AXPC1) [38]. This view shows known genes from RefSeq, EyeSAGE data, the candidate eye disease track (showing AXPC1), and ESTs from RPE/choroid and retina. Just overlapping the 5′ end of the mapped disease region position (corresponding to STS marker D1S2692) is a sequence for an EST from RPE/choroid [3] (CA396928) that shows exon/intron structure. This previously uncharacterized transcript has an open reading frame but no similarity with known proteins, suggesting it may be a non-coding RNA. This novel gene also has SAGE tag counts and, by SAGE, appears to be eye-preferred, suggesting it may be worthy of further investigation.

### Future plans

There is scope for many additions to NEIBank. EST analyses of eye tissues for additional species valuable to eye research (such as the 13-line ground squirrel whose cone-rich retina is an important model for human disease) are needed while even some human tissues such as normal cornea and conjunctiva are underrepresented in the databases. The rich resource of mouse genetic disease models needs to be assembled into a murine equivalent of the disease gene and candidate region databases, including an updated on-line resource for identifying knock-out and transgenic mice available for eye research. Integration of data from microarray experiments would be desirable, although insights into a useful way to do this are still needed. Expression data for miRNAs in eye tissues would be another useful addition. Meanwhile all the existing data needs regular curation to remain current.

### Conclusions

NEIBank provides cDNA clones and data from multiple eye tissues from many species. It combines all available data from eye EST projects and from SAGE analyses of human retina and RPE. In addition, it provides powerful tools for analyzing the transcriptional repertoire of the eye in humans and several model species. These expression data are further integrated with a comprehensive collation of human genetic eye disease information. Curation of the data and development of new informatics tools are ongoing processes, particularly as new genome resources arise and as formats and contents of other linked databases, such as GenBank and Unigene, evolve. Comments, queries, and additions from the community are encouraged.
